# Elevated Nitrite/Nitrate Ratio as a Potential Biomarker for the Differential Diagnosis of Pleural Effusions

**DOI:** 10.3390/antiox11071327

**Published:** 2022-07-06

**Authors:** Mu-Rong Chao, Yuan-Jhe Chang, Ying-Ming Shih, Jian-Lian Chen, Cheng-Chieh Yen, Chiung-Wen Hu

**Affiliations:** 1 Department of Occupational Safety and Health, Chung Shan Medical University, Taichung 402, Taiwan; mrchao@csmu.edu.tw (M.-R.C.); jeffchang@csmu.edu.tw (Y.-J.C.); ycj@csmu.edu.tw (C.-C.Y.); 2 Department of Occupational Medicine, Chung Shan Medical University Hospital, Taichung 402, Taiwan; 3 Division of Chest Medicine, Department of Internal Medicine, Changhua Christian Hospital, Changhua 500, Taiwan; kitofen@gmail.com; 4 School of Pharmacy, China Medical University, Taichung 404, Taiwan; cjl@mail.cmu.edu.tw; 5 Department of Public Health, Chung Shan Medical University, Taichung 402, Taiwan; 6 Department of Family and Community Medicine, Chung Shan Medical University Hospital, Taichung 402, Taiwan

**Keywords:** nitrite, nitrate, exudates, transudates, infection, LC-MS/MS

## Abstract

Pleural effusions (PEs) are common in clinical practice and can be due to many different underlying diseases such as cancer, congestive heart failure, or pneumonia. An accurate differential diagnostic categorization is essential, as the treatment and prognosis of PEs largely depend on its cause. In this study, we tested the hypothesis that nitrite and nitrate concentrations in PEs are associated with the inflammation and infection conditions. We therefore measured the nitrite and nitrate levels in 143 PE samples using a sensitive liquid chromatography-tandem mass spectrometry method and investigated their diagnostic potential in differentiating PEs. The results showed that nitrite concentrations and nitrite/nitrate ratios were higher in exudates than in transudates (NO_2_^−^: 2.12 vs. 1.49 μM; NO_2_^−^/NO_3_^−^: 23.3 vs. 14.0). Both the nitrite concentrations and the nitrite/nitrate ratios were positively correlated with the three Light’s criteria. Moreover, the receiver operating characteristic curve analysis revealed that the nitrite/nitrate ratio with an area under the curve of 0.71 could be a potential diagnostic biomarker in separating infectious PEs (IPEs) from other types of PEs. Taken together, the nitrite/nitrate ratio not only reflected the statuses of inflammation, but also the nitrate reduction by pathogenic bacteria infection in the pleural cavity. The nitrite/nitrate ratio could be a better biomarker in the differential diagnosis of PEs than the nitrite concentration alone.

## 1. Introduction

Pleural effusions (PEs), the accumulation of fluid in the pleural space, are a common clinical problem, resulting from pathologies that affect the pleural space such as congestive heart failure, malignancy, and infection (e.g., pneumonia). PE has been classified into exudates and transudates, based on the mechanism of fluid formation. Exudates result from the inflammation of the pleura or decreased lymphatic drainage, whereas transudates are the result of an imbalance in oncotic and hydrostatic pressures [1]. Light’s criteria have been used for more than 40 years and represent the diagnostic gold standard for differentiation between exudates and transudates. According to Light’s criteria, a fluid can the defined as an exudate if (i) it has a PE/serum protein ratio >0.5; (ii) a PE/serum lactate dehydrogenase (LDH) ratio >0.6; or (iii) a PE LDH level >2/3 the upper limits of the normal [2]. Despite the usefulness of Light’s criteria in identifying exudates, it has been reported that Light’s criteria misclassified about 20–30% of transudates as exudates [3], especially when patients received diuretics. Recently, several studies have proposed other biomarkers for helping and improving the differentiation of exudates and transudates such as total adenosine deaminase [4], cholesterol [5], and C-reactive protein [6].

Among the exudates, a major challenge in the diagnosis and management of PEs remains the differentiation between infectious pleural effusion (IPE, i.e., parapneumonic effusion or empyema) and malignant pleural effusion (MPE). The delay in the diagnosis and the initiation of appropriate therapy for IPE can increase the rate of complications [7]. The present Light’s criteria do not reliably identify an infectious etiology. The diagnosis of IPE often requires an invasive procedure (i.e., thoracentesis) to confirm the presence of infection. However, it has been reported that only a 60% of IPE has a positive culture result [3,7,8], and the time required (at least 5–7 days) to obtain the positive results prolongs the diagnosis and treatment. Although the pleural pH (<7.2) and glucose levels (<60 mg/dL) have previously been used as indicators for pleural drainage in the complicated parapneumonic effusions, these biomarkers are not sufficiently sensitive and lack specificity for infection [9]. The pleural white blood cell (WBC) count has also been applied to help diagnosis parapneumonic effusions and empyema (e.g., WBC > 10,000 cells/μL) [10]. However, because the pleural WBCs often have a wide range (500–50,000 cells/μL), the threshold of WBCs is not diagnostic.

An infection can cause inflammation. Nitric oxide (NO) is recognized as an important mediator and regulator of inflammatory responses [11], which is primarily produced by inducible nitric oxide synthase (iNOS) in inflammatory cells such as macrophages. Because the lifespan of NO is extremely short in humans, its major metabolites, nitrite and nitrate, have attracted significant attention in recent decades [12]. Since previous studies have shown that nitrite rather than nitrate reflects the regional endothelial iNOS activity during inflammation [13], and nitrite is less influenced by diet than nitrate [14,15], the nitrite level alone or the ratio of nitrite/nitrate in biological samples has been evaluated for its potential in clinical diagnosis. Elevated levels of nitrite in sputum were observed in patients with chronic obstructive pulmonary disease [16] or in the breath condensates of children with respiratory disease [17]. Most recently, an attempt has been made to use serum nitrite levels and nitrite/nitrate ratios as potential biomarkers for post-COVID-19 complications [18]. Compared with the nitrite and nitrate levels in blood (as reviewed in [19,20]), the origin and biosignificance of nitrite and nitrate in PEs have been rarely investigated in the literature.

Several studies have reported that the most common bacterial causes of parapneumonic effusion or empyema include the *Streptococcus milleri* group, *Streptococcus pneumoniae,* and *Staphylococcus aureus* [21]. Many infectious microorganisms can reduce nitrate to nitrite [22,23,24], which further inspired us to hypothesize whether the nitrite concentration and/or nitrite/nitrate ratio in PEs could help with the IPE diagnosis. We have previously demonstrated that the urinary nitrite concentration alone or the nitrite/nitrate ratio had satisfactory diagnostic potentials in screening patients with urinary tract infections (UTIs) (e.g., nitrite/nitrate ratio sensitivity: 95% and specificity: 91%) [25].

In this study, a total of 143 PE samples were collected and measured for the nitrite and nitrate concentrations using a sensitive and validated isotope-dilution LC-MS/MS method. The aim of the present study was to investigate whether the nitrite concentration and/or nitrite/nitrate ratio increased in the exudates and IPEs and to evaluate their diagnostic potential in helping to differentiate between IPEs and other types of PEs.

## 2. Materials and Methods

### 2.1. Chemicals

Sodium nitrate (NaNO_3_), sodium nitrite (NaNO_2_), 2,3-diaminonaphthalene (DAN, the derivatizing agent), nitrate reductase (from *Aspergillus niger*), β-nicotinamide adenine dinucleotide 2′-phosphate reduced tetrasodium salt hydrate (β-NADPH), and flavin adenine dinucleotide disodium salt hydrate (FAD) were obtained from Sigma–Aldrich; ^15^N-NaNO_2_ and ^15^N-NaNO_3_ were purchased from Cambridge Isotope Laboratories.

Standard stock solutions of nitrite and nitrate used to establish calibration curves were individually prepared by dissolving NaNO_2_ or NaNO_3_ in deionized water to the desired concentrations. The linear range for NO_2_^−^ was 0.03 to 2.0 μM, and each calibrator contained 0.5 nmol of the stable isotope-labeled internal standard (SIL-IS) ^15^N-NO_2_^−^. The linear range for NO_3_^−^ was 15.6 to 1000 μM, and each calibrator contained 6.25 nmol of the SIL-IS ^15^N-NO_3_^−^. The calibrators were processed and analyzed as described later for the PE samples.

### 2.2. Pleural Effusions Collection

This study was approved by both the Institutional Review Boards of Changhua Christian Hospital (CCH IRB No. 160621) and Chung Shan Medical University Hospital (CSMUH No. 10005) in Taiwan. All of the participants with clinical suspicion of pleural effusion taken by chest X-ray were adults aged over 18 during outpatient clinic visits or admission. Written, informed consent was obtained from the participants themselves, prior to enrollment. Pleural effusion was collected using ultrasound-guided thoracentesis by an attending physician. The discrimination of an exudate or transudate effusion was based on Light’s criteria [26]: (i) a PE/serum protein ratio >0.5; (ii) a PE/serum lactate dehydrogenase (LDH) ratio >0.6; or (iii) a PE LDH level >2/3 the upper limits of the laboratory’s reference range of serum LDH, either of which was considered exudate PE. The final diagnosis of PE was obtained from chart records. Malignant pleural effusion is defined as the malignant cells seen in effusion samples. Parapneumonic effusion means patients had neutrophil-predominant PE in a clinical scenario or when patients responded to antibiotic treatment [9,10], while empyema is defined as a positive bacterial culture result. Congestive heart failure (CHF) was confirmed by heart echocardiogram with a left ventricle ejection fraction (LVEF) less than 40%. Liver cirrhosis was diagnosed by liver sonography. Hypoalbuminemia was defined as serum albumin level less than 3 g per deciliter (g/dL). Chronic kidney disease (CKD) presented patients with a glomerular filtration rate (GFR) of less than 30 mL/min. Pulmonary tuberculosis was excluded from this study.

### 2.3. Analysis of Nitrite/Nitrate in Pleural Effusions Using Isotope-Dilution LC-MS/MS

**Figure 1:**
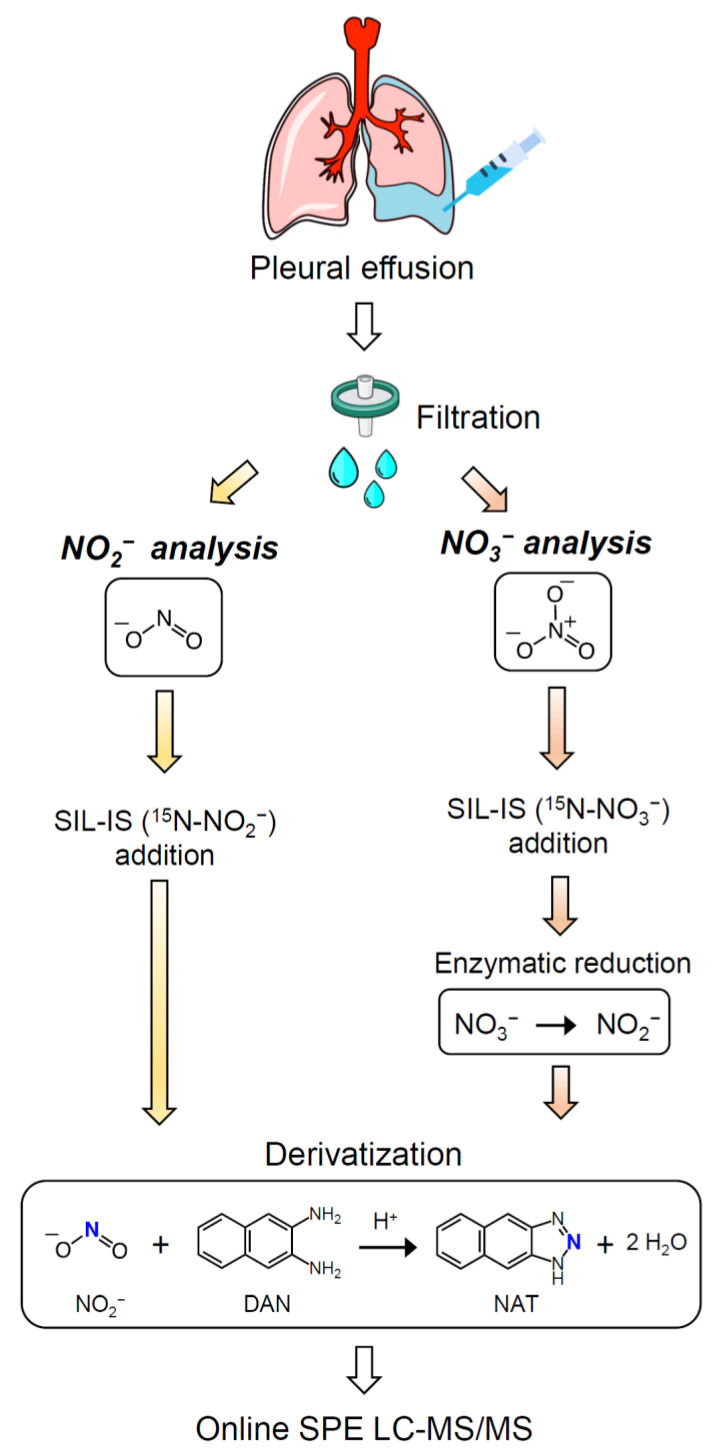
The sample preparation of PE for the nitrite and nitrate analysis using online SPE LC-MS/MS.

Concentrations of nitrite and nitrate in PE were measured by an isotope-dilution online solid phase extraction (SPE) LC-MS/MS method following chemical derivatization with DAN, as previously described by Chao et al. [25]. The analysis relies on the measurement of 2,3-naphthotriazole (NAT, the nitrite-DAN derivative). A scheme of the PE sample preparation is shown in Figure 1. Briefly, the PE sample was initially filtered using a 0.45 µm nylon filter. For nitrite analysis, 50 μL of filtrate was diluted five times with an aqueous solution containing 0.5 nmol of SIL-IS ^15^N-NO_2_^−^. The diluted PE sample was then derivatized with DAN at 37 °C for 30 min to yield NAT and ^15^N-NAT, followed by the addition of NaOH to terminate the reaction. The resulting reaction mixture was diluted 10 times with 10% (*v*/*v*) methanol containing 1 mM ammonium acetate, at which point the mixture was ready for online SPE LC-MS/MS analysis. To measure the nitrate level, it was converted to nitrite by nitrate reductase, followed by the derivatization of nitrite with DAN to form NAT [25]. Twenty-five μL of the filtrate was added to 25 μL of an aqueous solution containing 6.25 nmol of the SIL-IS ^15^N-NO_3_^−^, and the resultant mixture was incubated at room temperature for 60 min with nitrate reductase to reduce NO_3_^−^ to NO_2_^−^. To continue the derivatization reaction, the mixture containing the total nitrite (initial nitrite plus nitrite reduced from nitrate) was then diluted 20 times with deionized water and processed as described for nitrite. The nitrate concentration was calculated by subtracting the initial nitrite concentration from the total nitrite concentration.

**Figure 2:**
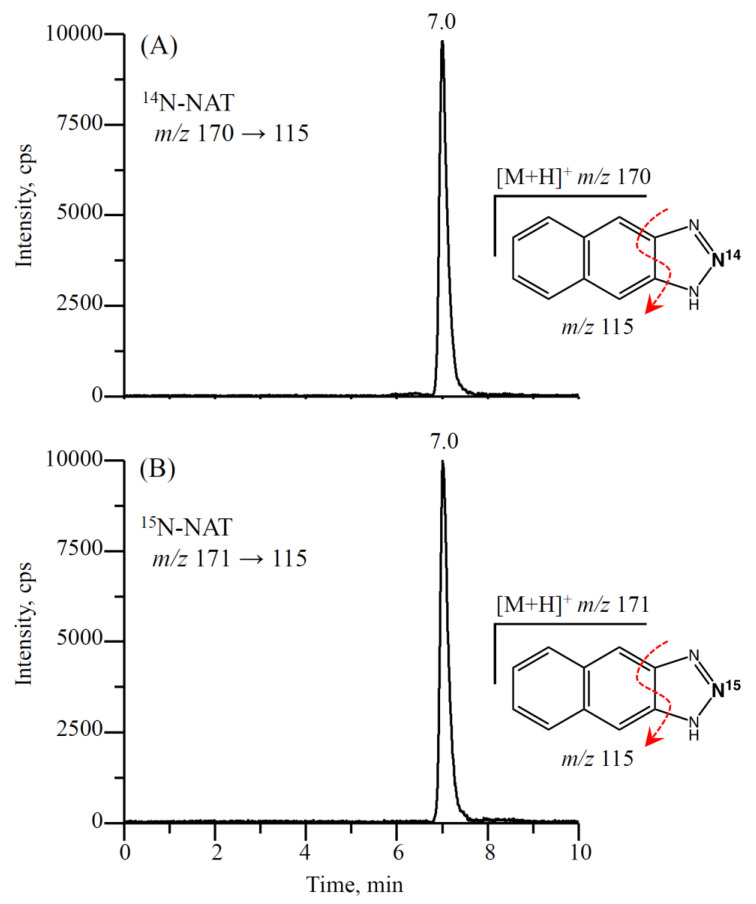
Chromatograms of the NAT (the nitrite-DAN derivate) in an exudate effusion, as measured by LC-MS/MS coupled with online SPE. MRM transitions: (**A**) *m*/*z* 170 → 115 for NAT and (**B**) *m*/*z* 171 → 115 for 15N-NAT in the positive mode.

NAT (the nitrite-DAN derivative) analysis was performed using an Agilent 1100 series HPLC system (Agilent Technologies, Germany) interfaced with an API 4000 QTrap hybrid triple quadrupole linear ion trap mass spectrometer (AB SCIEX, MA, USA) equipped with a TurboIonSpray source. The samples were analyzed in the positive ion multiple reaction monitoring (MRM) mode, and the transitions monitored were *m*/*z* 170 → 115 for NAT and *m*/*z* 171 → 115 for ^15^N-NAT. The injection volume was 10 μL. Representative chromatograms of NAT obtained from an exudate effusion by online SPE LC-MS/MS are shown in Figure 2.

### 2.4. Statistical Methods

The arithmetic mean and standard deviation (SD) were applied to describe the distributions of the demographical data. Geometric mean (GM) and 95% confidence interval (CI) were used to describe the PE parameters (i.e., nitrite, nitrate, nitrite/nitrate ratio, LDH, protein, etc.) due to the non-normal distributions. The Mann–Whitney U test was used to assess the differences among groups. The Spearman’s rank correlation coefficient was used to assess the correlation between the variables. The data were analyzed using IBM SPSS Statistics software v22 (IBM Corporation, Armonk, NY, USA).

## 3. Results

### 3.1. Etiology of PEs and General Characteristics of Patients

One hundred and forty-three patients were collected, and the detailed diagnosis of patients with effusion are summarized in Table 1. Of the 143 patients classified as having PEs, 83 (58%) were diagnosed with exudate effusion and 60 (42%) were diagnosed with transudate effusion. The exudate group, was further divided into two subgroups according to the diagnosis: malignant pleural effusion (MPE), 56 (39.2%) and infectious pleural effusion (IPE), 27 (18.9%). The transudate group was also further divided into four subgroups according to the cause of PE: CHF, 15 (10.5%); cirrhosis, 8 (5.6%); hypoalbuminemia, 33 (23.1%), and CKD, 4 (2.8%). The demographic data and PE characteristics of the 143 patients are summarized in Table 2. In general, the exudate group had a lower age than the transudate group (*p* = 0.004). Both groups had similar distributions of BMI and gender. All three of Light’s criteria and the total WBCs of the exudate group were significantly higher than those of the transudate group (*p* ≤ 0.001). No differences were observed in pH and the percentages of lymphocytes and neutrophils per total WBCs between the exudate group and transudate group.

### 3.2. Nitrite and Nitrate Concentrations in PEs

**Figure 3:**
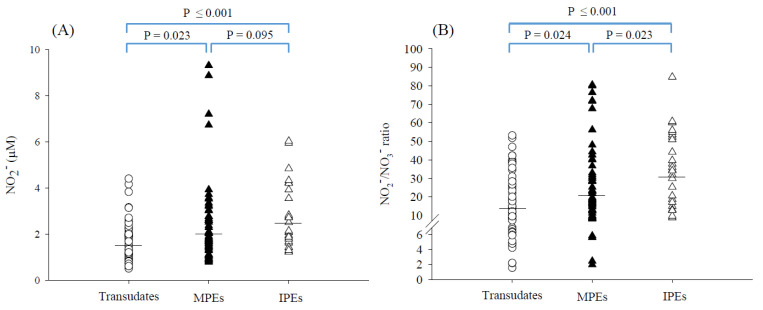
The distributions of (**A**) the nitrite concentrations and (**B**) nitrite/nitrate ratios (μM/mM) in transudates (*n* = 60), MPEs (*n* = 56), and IPEs (*n* = 27). The horizontal lines denote geometric means.

As shown in Table 2, the GM of nitrite concentration in the exudate group was found to be significantly higher than that in the transudate group (2.12 vs. 1.49 μM; *p* = 0.002). The GM of the nitrite/nitrate concentration ratio was significantly higher in the exudate group than in the transudate group (23.3 vs. 14.0; *p* ≤ 0.001). No significant difference in nitrate concentration was found between the exudate group and transudate group (*p* = 0.672). The nitrite concentrations and nitrite/nitrate concentration ratios were further compared between subgroups. Of the exudate group, the GM of nitrite concentration was higher in the IPEs than MPEs, but it did not reach statistical significance (2.45 vs. 1.99 μM, *p* = 0.095, see Figure 3A). However, the IPEs had significantly higher nitrite/nitrate ratios than the MPEs (GM of ratio: 30.5 vs. 20.6, *p* = 0.023, see Figure 3B). Of the transudate group, similarly, the nitrite concentrations were not statistically different among the subgroups, except for the PE caused by CHF. PE caused by CHF showed a higher GM of nitrite concentration than PE caused by hypoalbuminemia (1.82 vs. 1.31 μM, *p* = 0.035, Supplementary Figure S1A). Subsequently, for the nitrite/nitrate ratios, only PE caused by hypoalbuminemia showed a higher GM of ratio compared to the PE caused by cirrhosis (16.5 vs. 7.64, *p* = 0.027, Supplementary Figure S1B). There were no significant differences between other subgroups within the transudate group, probably because of the limited sample size of each subgroup.

**Figure 4:**
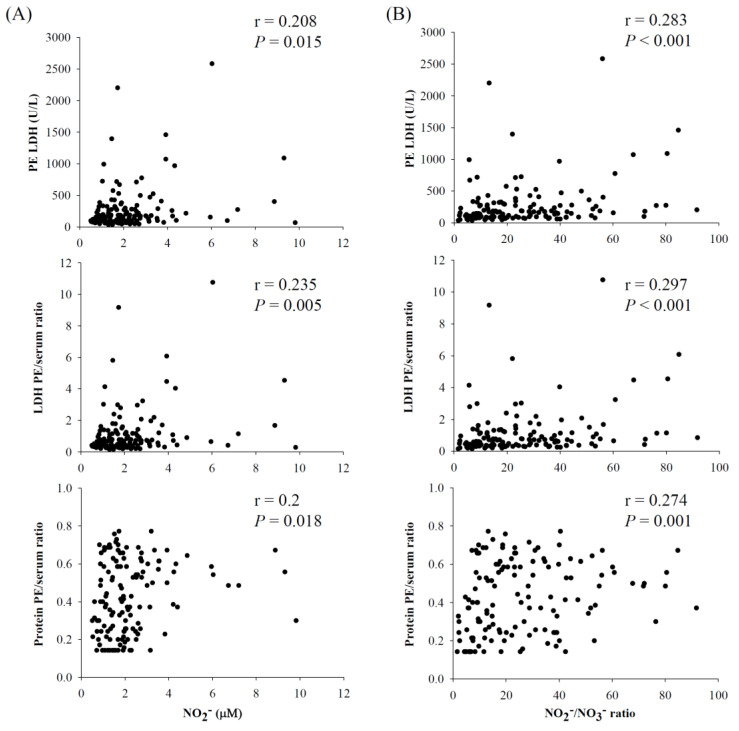
The correlations between Light’s criteria and (**A**) the nitrite concentrations or (**B**) nitrite/nitrate ratios. The correlation was estimated by the Spearman’s rank correlation coefficient.

Possible correlations between nitrite concentrations or nitrite/nitrate ratios, and the parameters of Light’s criteria were further assessed using the Spearman’s rank correlation coefficient. As shown in Figure 4, both nitrite concentrations (Figure 4A) and nitrite/nitrate ratios (Figure 4B) were positively and significantly correlated with each parameter of Light’s criteria as follows: nitrite and LDH level (r = 0.208, *p* = 0.015), nitrite and PE/serum LDH ratio (r = 0.235, *p* = 0.005), and nitrite and PE/serum protein ratio (r = 0.2, *p* = 0.018); nitrite/nitrate ratio and LDH level (r = 0.283, *p* < 0.001), nitrite/nitrate ratio and PE/serum LDH ratio (r = 0.297, *p* < 0.001), and nitrite/nitrate ratio and PE/serum protein ratio (r = 0.274, *p* = 0.001). Apparently, the nitrite/nitrate ratio had better correlations with the parameters of Light’s criteria than the nitrite concentration alone.

### 3.3. The Diagnostic Potential of Nitrite and Nitrate Concentrations in the Differentiation of PEs

**Figure 5:**
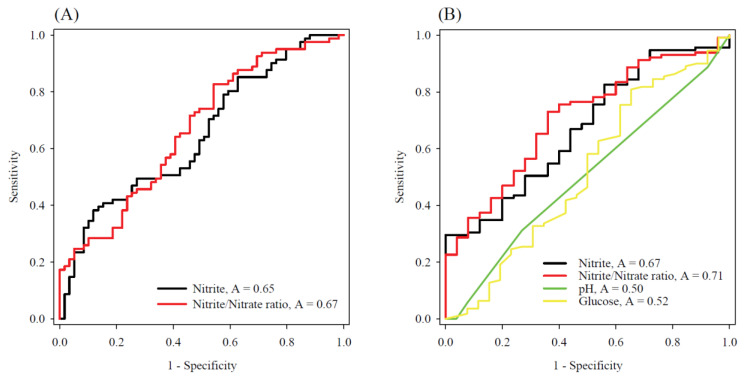
The ROC curve analysis of the nitrite concentrations and nitrite/nitrate ratios in PE to (**A**) differentiate between transudates and exudates, and (**B**) differentiating IPEs from other types of PEs (i.e., MPEs and transudates).

The diagnostic potential of the nitrite concentration and nitrite/nitrate ratio in discrimination between exudates and transudates were initially evaluated using the receiver operating characteristic (ROC) curve analysis. As shown in Figure 5A, the nitrite concentrations alone or the nitrite/nitrate ratios had nearly acceptable diagnostic accuracy, yielding an area under the curve (AUC) of 0.65 for nitrite concentration and 0.67 for the nitrite/nitrate ratio. However, when we assessed the diagnostic potential of the nitrite concentration and nitrite/nitrate ratio for identifying the IPE among all types of PEs, in particular, the nitrite/nitrate ratio showed a satisfactory diagnostic accuracy (AUC: 0.71, see Figure 5B) compared with the conventional diagnostic parameters of parapneumonic effusions (e.g., AUC: 0.50 for pH value and 0.52 for glucose level in PEs).

## 4. Discussion

Our study demonstrated, for the first time, (i) the distributions of nitrite and nitrate concentrations in different types of PEs; (ii) that the nitrite concentrations and nitrite/nitrate ratios in exudates were significantly higher than those in transudates; (iii) the nitrite concentrations and nitrite/nitrate ratios were positively correlated with the parameters of Light’s criteria; and (iv) IPEs had significantly higher nitrite/nitrate ratios than MPEs or transudates, showing a diagnostic potential in differentiating IPEs from other PEs.

To the best of our knowledge, this is the first isotope-dilution LC-MS/MS method to measure nitrite and nitrate in human PE samples. This LC-MS/MS method has been previously fully validated in human urine [25], and further successfully applied in PE samples in the present study. The LOD was estimated to be 0.003 μM for nitrite and 0.025 μM for nitrate. With this method, the nitrite concentrations in PEs were found to be 0.51–9.82 μM, while the nitrate concentrations were 0.023–0.838 mM. Because the nitrite concentrations in PEs were mainly less than 2 μM (92 out of 143 PEs samples, 64%), a high sensitivity of measurement is important to enable comprehensive PE measurement for research and clinical practices. Although the commercial dipstick for nitrite test has been widely applied in the clinical practice for the diagnosis of UTIs, the poor sensitivity of 2–8 μM [25,27] will lead to the lack of applicability in PE differentiation.

In the literature, very few studies have reported the nitrite concentration in PEs. Utine et al. [28] reported the nitrite concentration of 10.5–14.2 μM in the parapneumonic effusions of children by using a Griess colorimetric assay. The nitrite concentrations in the parapneumonic effusions of children were at least four times higher than our findings (GM: 2.45 μM for IPEs, see Figure 3A and Supplementary Table S1). This discrepancy could be partially attributed to the difference in the subject’s age (children vs. adults), infection condition, and the analytical method applied. The Griess colorimetric assay involving the spectrophotometric analysis of the azo dye obtained after reaction with the Griess reagent, has suffered from a low specificity in complex biological samples (e.g., blood and urine) due to the interferences with a number of endogenous and exogenous compounds (e.g., ascorbate, reduced thiols and phosphates [12,29,30]), which make nitrite measurements questionable.

The GM of the nitrite concentrations and nitrite/nitrate ratios were significantly higher in the exudates than in the transudates. Furthermore, both nitrite concentrations and nitrite/nitrate ratios were individually positively correlated with the three Light’s criteria. Light’s criteria reflect the consequences of local leaky capillaries and pleural inflammation due to infection or tumor. The capillary leaks secondary to inflammation result in a larger pleural pore-size [31], which allows the large-sized lipoproteins to accumulate in exudative pleural effusions. The elevated nitrite concentrations and nitrite/nitrate ratios observed in this study reflected the occurrence of pleural inflammation. Significant correlations between nitrite concentrations or nitrite/nitrate ratios and Light’s criteria could highlight the applicability of nitrite or nitrite/nitrate ratios in the differentiation of PEs (i.e., exudates vs. transudates).

It is of interest that the IPEs had particularly higher nitrite concentrations or nitrite/nitrate ratios than MPEs and transudates (see Figure 3). The production of nitrite in IPEs may arise from locally produced NO resulting from inflammation caused by infection [32], as evidenced by a neutrophil-dominant PE in the clinical scenario (neutrophils, GM %: 21% for parapneumonic effusions and 33% for empyema). It is well-known that neutrophils are normally the first responders to inflammation [33]. Neutrophils can kill invading pathogens by enhancing reactive oxygen species (ROS) and NO generation. Nitrite (or together with nitrate) has previously been used as a marker of NO formation in tissues and blood, and were found to be increased in various inflammation-associated diseases [13,34,35,36]. Elevated levels of sputum nitrite and nitrate have also been observed in patients with cystic fibrosis during acute pulmonary infection, suggesting an activation of iNOS in cystic fibrosis [37]. Furthermore, IPEs identified in this study were based on the pulmonary infiltrate responsive to antibiotic treatment or the positive culture test showing the presence of one or more microbial species such as *Streptococcus spp.*, *Staphylococcus aureus*, *Klebsiella oxytocain*, and *Actinomyces odontolyticus*, as observed in the present study (see Supplementary Table S1). These bacteria are a common cause of parapneumonic effusion and empyema [38]. Interestingly, these bacteria have been shown to be capable of reducing nitrate to nitrite [22,23,24] and could also increase the nitrite concentrations and nitrite/nitrate ratios.

The ROC curve analysis further showed a satisfactory result for the nitrite/nitrate ratio, with an area under the curve of 0.71 compared to the classical biomarkers (see Figure 5B), implying a superior diagnostic potential in separating IPEs from other types of PEs. Surprisingly, although the pH < 7.20, glucose < 60 mg/dL, or total WBCs > 10,000 cells/μL in PEs have previously been proposed to examine pleural infection, in this study only one out of 27 IPEs (~3.7 %) had a pH value <7.2, three out of 27 IPEs (~11%) had a glucose level <60 mg/dL, and two out of 27 IPEs (~7.4%) had a total WBCs >10,000 cells/μL. A cutoff value of ~31.5 for the nitrite/nitrate ratio might be adequate for the screening of IPEs because this ratio provided a satisfactory specificity of 75% and an acceptable sensitivity of 60%, which warrants further validation with larger sample size. Meanwhile, it was also noted that the nitrite concentration alone did not show a satisfactory diagnostic potential as the nitrite/nitrate ratio, as revealed by the ROC curve analysis. This is partially because the “nitrate” as the substrate of the pathogenic bacteria had a wide range of concentrations in the PE samples (i.e., 0.023–0.838 mM), which could highly influence the nitrite concentrations and thereby hamper the differential efficiency of the nitrite concentration in separating IPEs from other types of PEs. Alternatively, the nitrite/nitrate ratio reflects the percentage of nitrate reduction by pathogenic bacteria, which may help improve the diagnostic accuracy of IPEs, and may enhance the MPE diagnosis by excluding the IPEs among the exudates.

Some limitations of this study should be mentioned for consideration in future research. One is the small sample size for the PE samples, empyema in particular, which could affect the statistical significance of a successful diagnostic classification. Second, the inflammation-associated markers (e.g., C-reactive protein, 3-nitrotyrosine and 8-nitroguanine) could be included to further investigate the putative implication of microbial infection-induced inflammation. Third, the elevated nitrite concentrations and nitrite/nitrate ratios associated with infection could be further substantiated by spiking the ^15^N-labeled nitrate into the IPEs and tracing the microbial transformation of ^15^N-labeled nitrate to ^15^N-labeled nitrite using a ^15^N-labeled nitrite/nitrate tracer analysis by LC-MS/MS [15]. Finally, since nitrate is the denominator term in calculating the nitrite/nitrate ratio, a high dietary intake of nitrate might reduce the ratio and requires further investigation. In combination with urinary nitrate analysis [15], it could help to elucidate the dietary influence further.

## 5. Conclusions

In conclusion, the diagnosis of the IPEs can be difficult using the culture test because the pathogenic bacteria fails to be cultured for over 40% of IPEs. Indeed, only four out of 27 IPEs (~15%) had a positive culture in the present study. We therefore evaluated the diagnostic potential of nitrite and nitrate concentrations in PEs using a sensitive and specific LC-MS/MS method. The present method can be easily performed in a clinical laboratory, especially in hospitals that already implement routine mass spectrometric analysis. The results could be available for diagnosis within 2–3 h. By measuring the nitrite and nitrate concentrations in PEs, we have proposed for the first time that the ratio of nitrite to nitrate in PEs may be a potential tool to screen for IPEs. Despite the higher instrument cost, our findings may help to improve the diagnosis of IPEs and prevent both time-consuming testing for bacterial infection and inappropriate antibiotic use.

## Figures and Tables

**Table 1:** The clinical and pathological diagnosis of patients with PE (*n* = 143).

Cause	All Patients, *n* (%)
*Exudates*	83 (58)
Malignant	56 (39.2)
Lung	34 (23.8)
Breast	11 (7.7)
Others	11 (7.7)
Infectious	27 (18.9)
Parapneumonic	23 (16.1)
Empyema	4 (2.8)
*Transudates*	60 (42)
CHF	15 (10.5)
Cirrhosis	8 (5.6)
Hypoalbuminemia	33 (23.1)
CKD	4 (2.8)

Abbreviations: CHF, congestive heart failure; CKD, chronic kidney disease.

**Table 2:** The demographic and laboratory characteristics of the study population (*n* = 143).

Variables	Exudates (*n* = 83)	Transudates (*n* = 60)	*p* Value
Age, yr	69 ± 13 (41–92) ^a^	75 ± 14 (42–98)	0.004
Sex, male/female, n	42/41	33/27	
BMI, kg/m^2^	23 ± 3.9 (14–34)	22 ± 4.2 (15–44)	0.119
Pleural effusions			
pH	7.5 (7.5–7.6) ^b^	7.6 (7.5–7.6)	0.272
LDH, U/L	313 (264–371)	85.7 (78.1–94)	≤0.001
Protein, g/dL	3.7 (3.5–4.0)	1.7 (1.6–1.9)	≤0.001
LDH PE/serum ratio	1.3 (1.1–1.6)	0.4 (0.3–0.4)	≤0.001
Protein PE/serum ratio	0.5 (0.5–0.6)	0.3 (0.2–0.3)	≤0.001
Glucose, mg/dL	102 (86–120)	139 (129–150)	0.022
WBCs, cells/μL	1082 (824–1421)	242 (177–332)	<0.001
Lymphocytes,%	38 (29–50)	51 (43–59)	0.998
Neutrophils, %	8 (6–11)	7 (5–10)	0.639
Monocytes, %	8 (6–9)	8 (6–10)	0.390
Nitrite, μM	2.12 (1.88–2.40)	1.49 (1.31–1.69)	0.002
Nitrate, mM	0.094 (0.082–0.107)	0.108 (0.088–0.134)	0.672
Nitrite/nitrate ratio (μM/mM)	23.3 (19.7–27.6)	14.0 (11.3–17.4)	≤0.001

^a^ Age and BMI are expressed as mean ± SD (range). ^b^ Variables for pleural fluids are expressed as geometric mean (95% CI).

## Data Availability

Data are contained within the article or Supplementary Materials.

## Supplementary Materials

The following supporting information can be downloaded at: https://www.mdpi.com/article/10.3390/antiox11071327/s1, Figure S1: Distributions of (A) nitrite concentration and (B) nitrite/nitrate ratio (μM/mM) among transudates; Table S1: Laboratory characteristics of the IPEs.

## Abbreviations

CHF: congestive heart failure; CKD: chronic kidney disease; ESI: electrospray ionization; IPE: infectious pleural effusion; LC-MS/MS: liquid chromatography-tandem mass spectrometry; LDH: lactate dehydrogenase; LOD: limit of detection; LOQ: limit of quantification; MPE: malignant pleural effusion; PE: pleural effusion; NO: nitric oxide; SPE: solid-phase extraction; WBC: white blood cell.
