# Supplementary material for: Elevated Nitrite/Nitrate Ratio as a Potential Biomarker for the Differential Diagnosis of Pleural Effusions
Source: Antioxidants (Basel). 2022 Jul 6;11(7):1327. doi: 10.3390/antiox11071327 (PMC9312090; doi:10.3390/antiox11071327)
Supplement: Supplementary file 1 [file antioxidants-11-01327-s001.zip › antioxidants-1738909-supplementary.pdf]

Supplementary Data

Elevated Nitrite/Nitrate Ratio as a Potential Biomarker for the Differential Diagnosis of Pleural Effusions

Mu-Rong Chao [1,2,†], Yuan-Jhe Chang [1], Ying-Ming Shih [3,†], Jian-Lian Chen [4], Cheng-Chieh Yen [1] and Chiung-Wen Hu [5,6,*]

Department of Occupational Safety and Health, Chung Shan Medical University, Taichung 402, Taiwan; mrchao@csmu.edu.tw (M.-R.C.); jeffchang@csmu.edu.tw (Y.-J.C.); ycj@csmu.edu.tw (C.-C.Y.)
[2] Department of Occupational Medicine, Chung Shan Medical University Hospital, Taichung 402, Taiwan
[3] Division of Chest Medicine, Department of Internal Medicine, Changhua Christian Hospital, Changhua 500, Taiwan; kitofen@gmail.com
[4] School of Pharmacy, China Medical University, Taichung 404, Taiwan; cjl@mail.cmu.edu.tw
[5] Department of Public Health, Chung Shan Medical University, Taichung 402, Taiwan
[6] Department of Family and Community Medicine, Chung Shan Medical University Hospital, Taichung 402, Taiwan
* Correspondence: cwhu0823@gmail.com or windyhu@csmu.edu.tw
† These authors contributed equally to this work.

Table S1. Laboratory characteristics of the IPEs.

**Variables**	**IPEs (*n* = 27)**[a]
pH	7.5 (7.5-7.6)[b]
LDH, U/L	286 (197-415)
Protein, g/dL	3.7 (3.2-4.2)
LDH PE/serum ratio	1.2 (0.8-1.7)
Protein PE/serum ratio	0.5 (0.5-0.6)
Glucose, mg/dL	106 (77-147)
WBCs, cells/μL	1660 (904-3050)
Lymphocytes,%	21 (11-39)
Neutrophils, %	22 (15-33)
Monocytes, %	7 (5-11)
Nitrite, μM	2.45 (2.03-2.96)
Nitrate, mM	0.088 (0.07-0.113)
Nitrite/nitrate ratio (μM/mM)	30.5 (24.1-38.7)

[a]Four out of 27 IPE samples were empyema. Multiple microbial species were observed: one IPE showed the existence of *actinomyces odontolyticus*, one IPE showed the existence of both *staphylococcus aureus* and *klebsiella oxytocain*, one IPE showed the existence of *streptococcus spp.*, and one showed the existence of both *streptococcus spp.* and *streptococcus constellatus*.

[b]Variables for pleural fluids are expressed as geometric mean (95% CI).

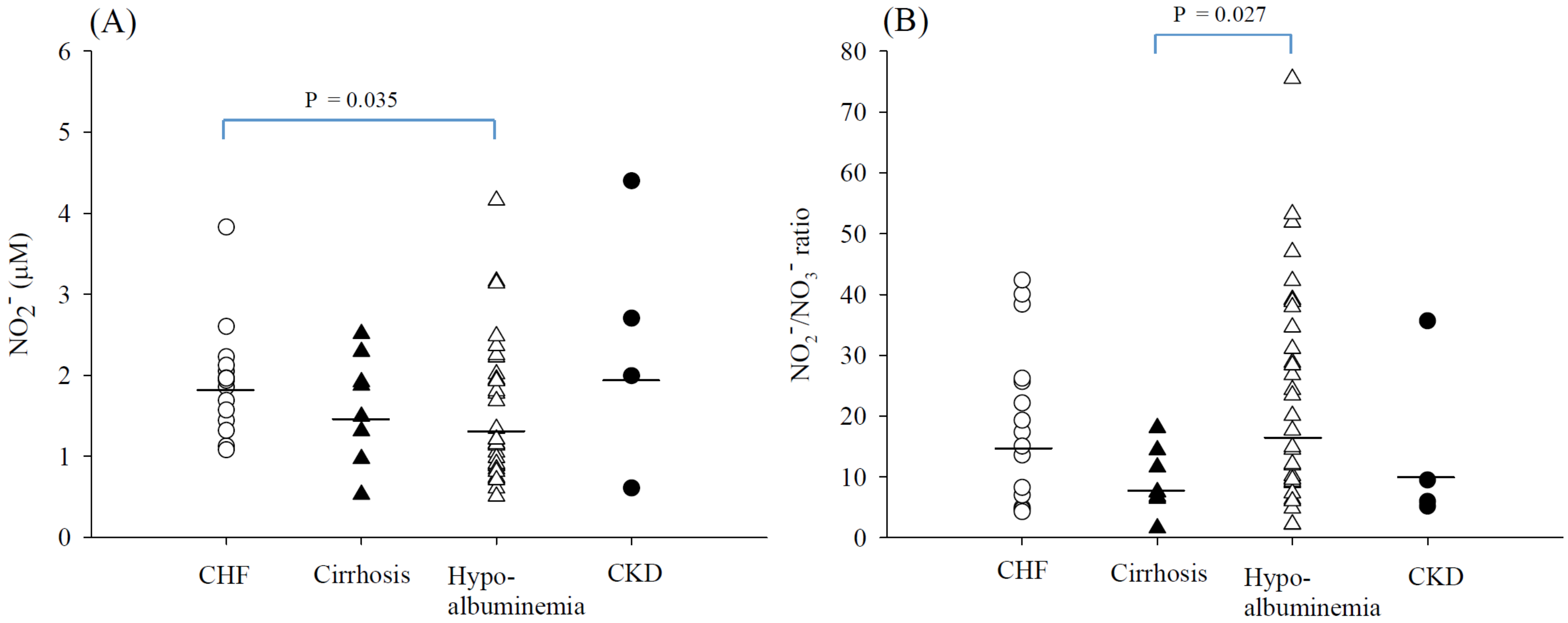

Figure S1. Distributions of (**A**) nitrite concentration and (**B**) nitrite/nitrate ratio (μM/mM) among transudates; CHF (*n* = 15), liver cirrhosis (*n* = 8), hypoalbuminemia (*n* = 33) and CKD (*n* = 4). The horizontal lines denote geometric means.
